# An unusual cause of a complete heart block in a young healthy man! (a case report)

**DOI:** 10.11604/pamj.2020.37.391.25927

**Published:** 2020-12-31

**Authors:** Salma Charfeddine, Syrine Triki, Wiem Feki, Tarak Ellouze, Leila Abid, Rania Hammami, Samir Kammoun

**Affiliations:** 1 Cardiology Department, Hedi Chaker University Hospital, Sfax, Tunisia,; 2 University of Medicine, Sfax, Tunisia,; 3 Radiology Department, Hedi Chaker University Hospital, Sfax, Tunisia

**Keywords:** Myocarditis, heart-block, high degree atrioventricular block, case report

## Abstract

Acute myocarditis represents a challenging diagnosis as there is no pathognomonic clinical presentation. It is rare to see heart block as the first-and-only presentation of infectious myocarditis. We report the case of a young healthy patient who presented with syncope secondary to a complete heart block. It was caused by acute presumed viral myocarditis. The diagnosis was confirmed with cardiac magnetic resonance imaging. With close monitoring, the EKG abnormalities resolved over the following 5 days. In this case report, we present the importance of several imaging tools to diagnose a rare and reversible cause of conduction disturbances. In at-risk individuals, clinicians should rule out a treatable cause of heart block before proceeding with permanent pacemaker implantation due to enormous clinical and cost implications involved.

## Introduction

Acute myocarditis represents a challenging diagnosis as there is no pathognomonic clinical presentation [1]. It ranges from asymptomatic infection to fulminant heart failure and sudden death. Various electrocardiographic (EKG) abnormalities have been reported in patients with myocarditis. It includes abnormalities in the ST-T wave segment, Q waves, atrioventricular block (AV block) or bundle branch blocks [2]. It is rare to see heart block as the first-and-only presentation of infectious myocarditis [3]. The diagnosis should be considered when physicians encounter a young patient with high-degree heart block associated to atypical symptoms of infection.

## Patient and observation

**Figure 1:**
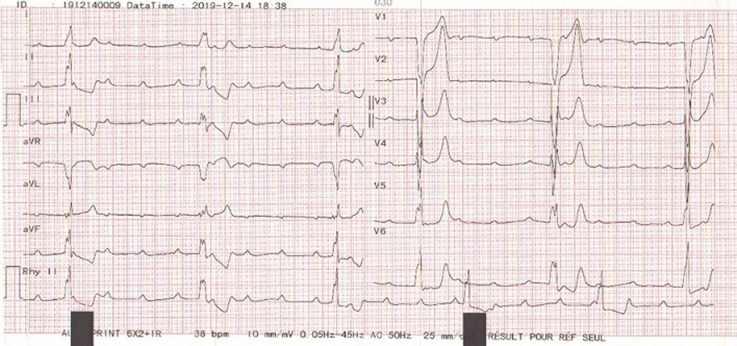
EKG showing heart block with complete atrioventricular dissociation, and idioventricular rhythm at 38 beats/minute

**Figure 2:**
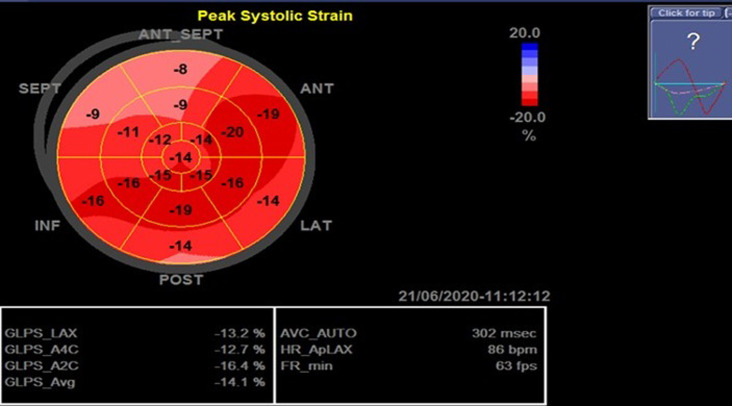
reduced left ventricular longitudinal strain (LV GLS -14.1%) with marked alteration in the anteroseptal segments

A 19-year-old healthy man was admitted to our cardiology department following a syncopal episode. His past medical history was unremarkable. He no family history of heart disease or sudden death. Two-days prior the syncope, he developed chest pain and an influenza-like illness consisting of fevers, rhinorrhea, and sore throat. At the physical exam, the patient was febrile at 39°C. He had an inflamed throat, bradycardia at cardiac auscultation and there were no signs of heart failure. The electrocardiogram (EKG) showed grade III atrioventricular (AV) bloc with a ventricular rate of 38 beats per minute (Figure 1). Laboratory studies were remarkable for a HS-troponin level 181ng/L, a white blood cell count of 14270/mm^3^ with 48% lymphocytes, and elevated C-reactive protein at 90mg/L. Serum electrolytes, kidney and liver functions were within normal limits. A chest X-ray showed an appropriate cardiac size. The initial transthoracic echocardiography (TTE) showed a normal global left ventricular function (left ventricular ejection fraction (LVEF) 60%) and wall motion abnormalities (anteroseptal hypokinesia), with reduced left ventricular global longitudinal strain (LV GLS= -14.1%) (Figure 2).

**Figure 3:**
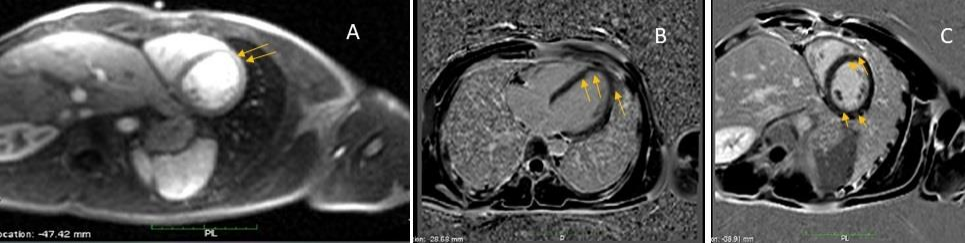
A) cardiac magnetic resonance (CMR) showed increased signal intensity at the mid-lateral wall on T2-weighted images; late enhancement revealed a patchy subepicardial hyperenhancement of the medio-distal segments of the anterior and anterolateral walls (B and C)

**Figure 4:**
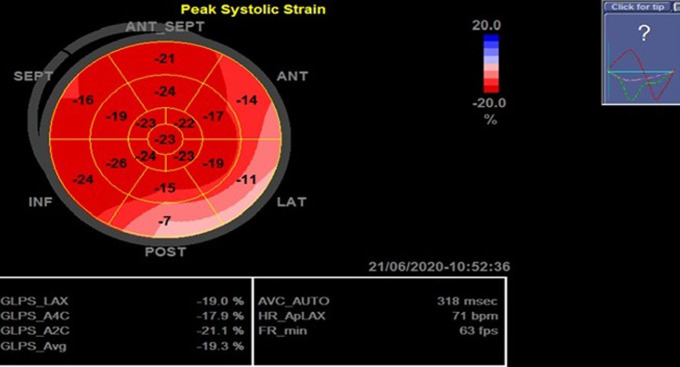
left ventricular longitudinal strain (LV GLS -19.3%) with mild alteration in the anterolateral segments

Cardiac magnetic resonance (CMR) showed increased signal intensity at the mid-lateral wall on T2-weighted images. Late enhancement revealed a patchy subepicardial hyperenhancement of the medio-distal segments of the anterior and anterolateral walls which confirm the diagnosis of myocarditis (Figure 3). The patient was treated with cefotaxime and doxycycline. All serological tests (chlamydia, coxiella, mycoplasma, rickettsia, lyme and bartonella) obtained during the hospitalization were negative. Five days later, the patient was asymptomatic. The 24 hours ECG monitoring showed a sinus rhythm. The TTE revealed a normal LVEF and an improvement in the left ventricular global longitudinal strain (LVGLS) with segmental reduced values concordant with the CMR abnormalities (Figure 4). At one-month follow-up the patient made a full recovery without any recurring cardiovascular symptoms or abnormal electrocardiographic or echocardiographic findings.

## Discussion

Acute myocarditis represents a challenging diagnosis as there is no pathognomonic clinical presentation [1]. The true incidence of infectious myocarditis in young healthy patients is unknown because of the frequently asymptomatic nature of infection. Several viruses are believed to account for the majority of cases, but a causative pathogen is identified in fewer than 10% of cases [4]. The EKG abnormalities are often early manifestations of acute myocarditis and may carry important clinical and prognostic implications [5]. Most EKGs demonstrate nonspecific and usually benign ST and T wave abnormalities, axis deviation, prolonged QRS duration, or premature ventricular contractions [6]. More serious arrhythmias or conductive disturbances such as AV conduction block, as seen in our patient, are estimated to occur in fewer than 20% of cases and are correlated to worse prognosis [6].

Common causes of acquired AV block that need to be ruled out when considering a diagnosis of myocarditis in young patient include ischemic heart disease and acute myocardial infarction, cardiomyopathies including hypertrophic obstructive cardiomyopathy, infiltrative myocardial processes (e.g. amyloidosis, sarcoidosis, hemochromatosis, lymphoma, multiple myeloma), hyperkalemia, collagen vascular disease (e.g. lupus, rheumatoid arthritis), increased vagal tone (due to pain, carotid sinus hypersensitivity, athletic training), thyroid disease, and drugs (beta blockers, calcium channel blockers, amiodarone, digitalis, adenosine, etc.) [6]. Most of these conditions will be apparent on history, or have readily observable manifestations that will help in pinpointing the diagnosis. Hence, it is apparent that the list of causes of acquired heart block in young healthy patients with no other manifestations is limited.

High-degree AV blocks have been related to higher morbidity and mortality in all patients with myocarditis [3]. The pathogenesis of conduction disturbances is influenced by pathogen tropism for cardiac myocytes and the inflammatory response of the host [7]. Viruses replicating within myocytes provoke an adaptive immune response characterized by the influx of natural killer cells and T cells. These cells are ultimately responsible for viral clearance, but in the process cause cytokine-mediated cellular damage and edema that result in interruption of the conduction pathways [8]. Clinically, these conduction disturbances may manifest diversely as AV block, bundle-branch and intraventricular block, or sinus arrest [6]. Pathological mechanisms for AV block, as seen in this case, are most often attributed to diffuse and severe inflammation in the right and left bundle branches, especially in the terminal portions [6]. The severity of pathological changes may reflect the degree and reversibility of AV block. The diagnosis of acute myocarditis is based on symptoms, electrocardiography, elevated myocardial biomarkers, and imaging [8]. The imaging methods such as echocardiography and CMR are useful tools to diagnose and monitor the progression of myocarditis, but the gold standard for diagnosis remains endomyocardial biopsy [8].

Echocardiography is useful as an initial diagnostic tool to detect the presence and severity of regional or global ventricular dysfunction and chamber dilation in children with acute viral myocarditis. Speckle tracking echocardiography is a quantitative, objective technique for intrinsic cardiac deformation in real time, providing accurate assessment of abnormal regional wall contractility as in myocarditis. Several studies have shown the superiority of strain imaging over the conventional methods used for assessment of regional myocardial dysfunction [9]. CMR imaging provides non-invasive tissue characterization of the myocardium and can support the diagnosis of myocarditis [10]. The CMR findings consistent with myocarditis should be based on Lake-Louise criteria [10]. In the setting of our patient, reduced segmental speckle tracking rate and CMR findings defined by regional myocardial signal intensity increase in T2-weighted oedema and Increased myocardial and subepicardial late gadolinium enhancement were consistent with myocardial inflammation.

## Conclusion

Complete heart block is usually an ominous finding that might require placement of a permanent pacemaker. Infectious myocarditis is a reversible cause of cardiac conduction abnormalities that should definitely be considered in the differential diagnosis of heart block before resorting to permanent pacemaker implantation, especially in young healthy patients. A suggestive history and physical exam, coupled with abnormal echocardiographic findings and positive CMR helps in the diagnosis.
